# Genetic and phenotypic landscape of the mitochondrial genome in the Japanese population

**DOI:** 10.1038/s42003-020-0812-9

**Published:** 2020-03-05

**Authors:** Kenichi Yamamoto, Saori Sakaue, Koichi Matsuda, Yoshinori Murakami, Yoichiro Kamatani, Keiichi Ozono, Yukihide Momozawa, Yukinori Okada

**Affiliations:** 1 Department of Statistical Genetics, Osaka University Graduate School of Medicine, Suita, 565-0871 Japan; 2 Department of Pediatrics, Osaka University Graduate School of Medicine, Suita, 565-0871 Japan; 3 Laboratory for Statistical Analysis, RIKEN Center for Integrative Medical Sciences, Yokohama, 230-0045 Japan; 4 Department of Allergy and Rheumatology, Graduate School of Medicine, The University of Tokyo, Tokyo, 113-8655 Japan; 5 Department of Computational Biology and Medical Sciences, Graduate School of Frontier Sciences, The University of Tokyo, Tokyo, 108-8639 Japan; 6 Division of Molecular Pathology, Institute of Medical Science, The University of Tokyo, Tokyo, 108-8639 Japan; 7 Laboratory of Complex Trait Genomics, Department of Computational Biology and Medical Sciences, Graduate School of Frontier Sciences, The University of Tokyo, Tokyo, 108-8639 Japan; 8 Laboratory for Genotyping Development, RIKEN Center for Integrative Medical Sciences, Yokohama, 230-0045 Japan; 9 Laboratory of Statistical Immunology, Immunology Frontier Research Center (WPI-IFReC), Osaka University, Suita, 565-0871 Japan; 10 Integrated Frontier Research for Medical Science Division, Institute for Open and Transdisciplinary Research Initiatives, Osaka University, Suita, 565-0871 Japan

**Keywords:** Machine learning, Next-generation sequencing, Genome-wide association studies

## Abstract

The genetic landscape of mitochondrial DNA (mtDNA) has been elusive. By analyzing mtDNA using the whole genome sequence (WGS) of Japanese individuals (*n* = 1928), we identified 2023 mtDNA variants and high-resolution haplogroups. Frequency spectra of the haplogroups were population-specific and were heterogeneous among geographic regions within Japan. Application of machine learning methods could finely classify the subjects corresponding to the high-digit mtDNA sub-haplogroups. mtDNA had distinct genetic structures from that of nuclear DNA (nDNA), characterized by no distance-dependent linkage disequilibrium decay, sparse tagging of common variants, and the existence of common haplotypes spanning the entire mtDNA. We did not detect any evidence of mtDNA–nDNA (or mtDNA copy number–nDNA) genotype associations. Together with WGS-based mtDNA variant imputation, we conducted a phenome-wide association study of 147,437 Japanese individuals with 99 clinical phenotypes. We observed pleiotropy of mtDNA genetic risk on the five late-onset human complex traits including creatine kinase (*P* = 1.7 × 10^−12^).

## Abstract (web summary)

Kenichi Yamamoto et al. report a genetic analysis of mitochondrial DNA (mtDNA) and a phenome-wide association study in Japanese individuals from the BioBank Japan Project. They describe the genetic landscape of the mitochondria and identify pleiotropic mtDNA variants associated with 5 late-onset complex traits.

## Introduction

Mitochondria, double-membrane cytoplasmic organelles, developed from the engulfment of an α-proteobacterium by a precursor of the modern eukaryotic cell 2.5 billion years ago, coevolved with the nucleus, and became specialized in generating energy^1^. Mitochondria produce energy essential for homeostasis of organs using oxidative phosphorylation (OXPHOS). Mitochondria also regulate intracellular calcium levels, apoptosis, and epigenetic mechanisms^2^. Mitochondria have their own DNA called mitochondrial DNA (mtDNA), which is a double-stranded circular DNA of ~16,569 base pair (bp), and encodes 37 genes that are related with OXPHOS (*n* = 13), transfer RNAs (*n* = 22), or ribosomal RNAs (*n* = 2). Hundreds of mitochondria reside in a single cell and each mitochondrion has several mtDNA molecules in itself. Furthermore, 1000–2000 mitochondria-related genes in nuclear DNA (nDNA) have been identified, which regulate gene expressions and mtDNA copy numbers (mtCNs) via interactions with mtDNA^3–6^.

A mutation rate in mtDNA is higher than in that of nDNA^2^. The footprint of accumulated variants (i.e., homoplasmy) represents the result of natural selection and genetic drift during the expansion of the human populations. Due to maternal inheritance and lack of recombination, mtDNA variants in a population are characterized as a set of mtDNA haplotypes, which are called haplogroups^1,2,7,8^. Reflecting essential biological roles, mtDNA variants are associated with risks of a variety of human complex traits such as metabolic diseases^9–11^, neurodegenerative diseases^12^, longevity^13,14^, and renal functions^15^.

Whereas many studies about mtDNA have been conducted to date, the genetics of mitochondria is still unclear. Next-generation sequencing enables us to examine human genome variants in detail, but few high-resolution analyses on mtDNA variants, haplogroups, and their differences between populations have been performed. As haplogroups have been defined based on historical accumulation of nomenclatures, it has been unclear how the defined haplogroups could reflect actual mtDNA diversity within a population. Although the difference of linkage disequilibrium (LD) structure between mtDNA and nDNA has been reported^16^, a comprehensive evaluation of mtDNA LD using whole genome sequence (WGS) data has not been conducted, especially in non-European populations. The details of the mtDNA–nDNA interactions are also still unknown. Considering population specificity of mtDNA haplotype structure, phenome-wide association study (PheWAS) utilizing a large-scale biobank cohort with the adjustment for population structure is required to robustly identify mtDNA variants associated with human complex traits. However, few studies have been conducted, especially in non-European population^17^.

Here we provide a comprehensive analysis that characterizes the genetic and phenotypic landscape of mitochondria in the Japanese population. (i) We constructed a high-resolution mtDNA variant list and haplotype classifications in the Japanese population based on deep WGS of ~2000 individuals. (ii) We conducted unsupervised clustering of the mtDNA variant patterns and assessed their correspondence with defined haplogroups by using a set of machine learning (ML) methods. (iii) We quantitatively assessed positional distributions and LD structure of the mtDNA variants by parallelly comparing with those of nDNA. (iv) We performed genome-wide scans to investigate the mtDNA–nDNA and mtCN–nDNA genotype associations. (v) Finally, we conducted mitochondrial genome-wide genotype imputation of genome-wide association study (GWAS) data of more than 140,000 Japanese individuals, by using the population-specific WGS reference data. We then conducted a PheWAS of 99 complex human disease and quantitative traits.

## Results

### High-resolution mtDNA variant and haplogroup lists in the Japanese population

We re-analyzed the previously reported WGS data of the Japanese population (*n* = 1928)^18^. We realigned the WGS reads on the human reference genome GRCh37, which includes the revised Cambridge Reference Sequence (rCRS, NC_012920.1) as the human reference mitochondrial genome. The rCRS has been widely used in mitochondrial genome analyses including Japanese individuals. We only used the reads uniquely mapped on the mitochondrial region to avoid the contamination of nuclear copies of the mitochondrial genome (nuMTs)^19^. In this study, we focused on the analyses of homoplasmy. Then, we identified 2023 variant sites, of which 63 sites were multiallelic (the mean depth = 1488; Supplementary Data 1). Of these, 516 variants (25.5%) were newly identified in our WGS data. Minor allele frequency (MAF) spectra indicated that the majority of the identified variants were rare in Japanese; rare variants (MAF < 0.5%), low-frequency variants (0.5% ≤ MAF < 5%), and common variants (MAF ≥ 5%) accounted for 79.3%, 16.4%, and 4.3%, respectively (Supplementary Fig. 1). We observed clear concordances of the alternative allele frequencies with those in the previously reported two Japanese databases (1507 and 1025 variants with 3.5KJPN [*n* = 3552] and Giib-JST mtSNP [*n* = 672], respectively; Supplementary Fig. 2)^7,20,21^. As previously reported^22,23^, mutational spectrum indicated a high transition to transversion (Ti/Tv) ratio of 16.44 (Supplementary Fig. 3).

**Fig. 1:**
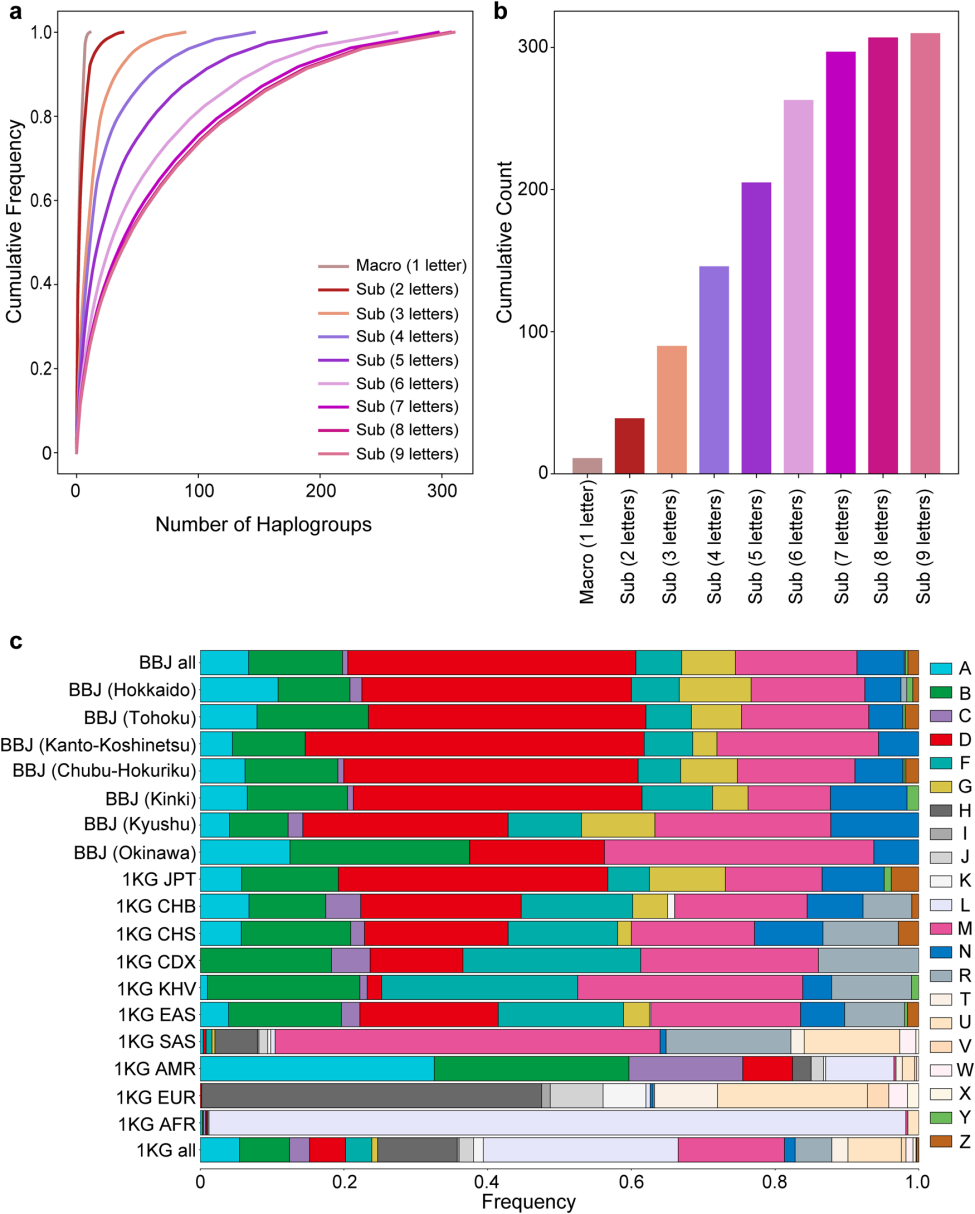
High-resolution spectra of mtDNA haplogroups in the Japanese population. The detailed haplogroup distributions based on the Japanese WGS data (*n* = 1928). **a** Cumulative frequency spectra of the haplogroups from macrohaplogroup (one letter) to sub-haplogroups (nine letters). **b** Cumulative counts of the haplogroups from macrohaplogroup (one letter) to sub-haplogroup (nine letters). **c** Stacked bar plots of the frequencies of the macrohaplogroups within the geographical regions in Japan and subpopulations from 1KG. The geographical regions in Japan are defined as Hokkaido, Tohoku, Kanto-Koshinetsu, Chubu-Hokuriku, Kinki, Kyushu, and Okinawa from northeast to southwest areas of Japan, as described elsewhere^42^.

Next, each individual was classified into the mtDNA haplogroup based on a variant list detected by the WGS using HaploGrep (v2.1.14)^24^. Haplogroups are classifications of the mtDNA haplotypes defined according to a set of the specific mtDNA variants. As mtDNA is a haploid genome, the detected variants could be directly used for haplogroup classification without phasing. Nomenclature of each haplogroup is hierarchically defined based on the number of the letters (from one to nine), which was divided into sub-haplogroups (e.g, “D4b” as three letters). The number of the haplogroups monotonically increased from the macrohaplogroup (*n* = 11 at one letter) to the sub-haplogroups with larger number of letters (*n* = 310 at nine letters; Supplementary Data 2). Increments in the number of the haplogroups became limited from seven to nine letters (Fig. 1a, b).

The frequency distribution of each haplogroup was obtained according to geographical regions in Japan (as defined by BioBank Japan Project: Hokkaido, Tohoku, Kanto-Koshinetsu, Chubu-Hokuriku, Kinki, Kyushu, and Okinawa from northeast to southwest areas of Japan; Supplementary Fig. 4)^18^ and all the populations from the 1000 Genomes Project Phase 3 (1KG, *n* = 2504; Fig. 1c)^25^. In the Japanese population, macrohaplogroups A, B, D, M, and N had more than 1% of frequencies across all the regions. In the regions from Hokkaido to Kyushu, macrohaplogroup D was most prevalent (>28%), followed by M and B. In contrast, the different spectrum was observed in Okinawa (the islandic region at the most southwest area in Japan), where M and B were more prevalent (37.5% and 25%, respectively) than D (18.8%). Furthermore, although the D4a and D4b haplogroups were prevalent from Hokkaido to Kyushu, the D4c haplogroup was prevalent in Okinawa (Supplementary Fig. 5). Although the haplogroup spectra in 1KG East Asians (EAS) were relatively similar to those in Japanese, R was more enriched in 1KG EAS, and D, G, and M were more frequent in Japanese. 1KG populations other than EAS showed distinct haplogroup patterns from the Japanese population. M, A, H, and L were most prevalent in 1KG South Asians, Americans, Europeans, and Africans (AFR), respectively. Especially, 1KG AFR showed the least diversity within macrohaplogroups, of which African-specific macrohaplogroup of L accounted for >90% of frequencies.

### Unsupervised ML approaches deconvoluted mtDNA classification patterns

To evaluate how the defined haplogroups reflect the mtDNA diversity within a population, we conducted unsupervised clustering of the subjects based on the mtDNA variants and evaluated the concordances with haplogroup assignments. We adopted three unsupervised ML classification approaches of phylogenetic approach, principal component analysis (PCA), and uniform manifold approximation and projection for dimensionality reduction (UMAP).

**Fig. 2:**
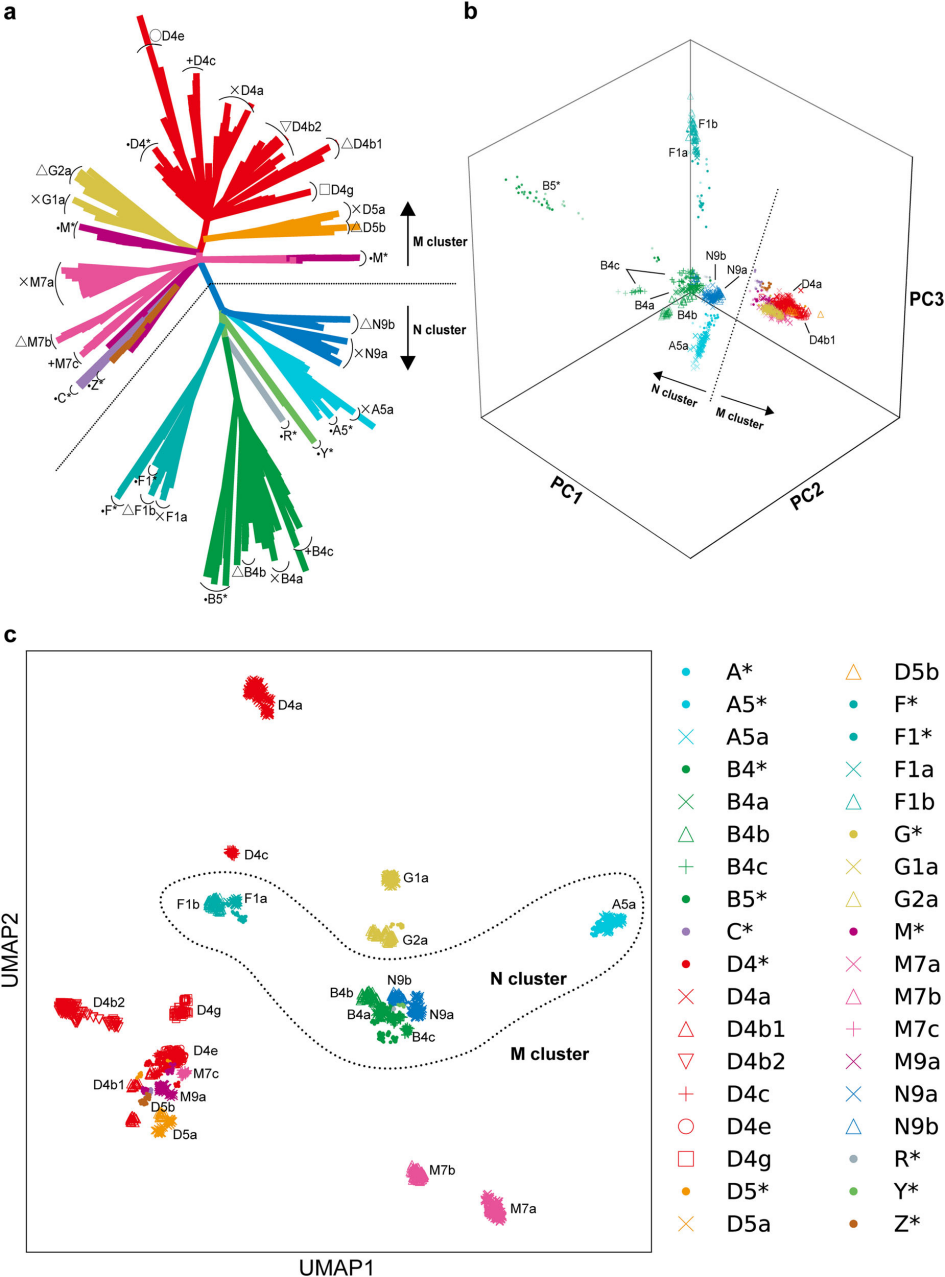
Unsupervised ML-based sample classification of the Japanese mtDNA variant data. All the three unsupervised ML method classifications were performed on the WGS variant data of the Japanese population (*n* = 1928). **a** The unrooted phylogenetic tree using maximum-parsimony method. **b** The 3D plot of the top three components of PCA. **c** The plot of the two components of UMAP. Each color and marker represents haplogroups. Distinction between the M and N haplogroup clusters is displayed with dotted lines in each panel.

We first constructed the phylogenetic tree of the WGS individuals, which was illustrated as an unrooted tree type (Fig. 2a). The tree branch was mainly divided into the major two lineages at the root base, which were known as the “M” and “N” clusters. Each major lineage was further divided into sub-lineages corresponding to the sub-haplogroups. Next, we applied the linear dimensionality reduction technique of PCA and examined up to the 20 PCs. The explained variances were 12.6% for the top 20 PCs. As the two-dimensional plot of the PC1 and PC2 was difficult to fully capture the cluster classifications (Supplementary Fig. 6), we adopted the three-dimensional plot consisting of the top three PCs (Fig. 2b). The major M and N clusters were also illustrated as distinct groups in the PCA plot. In contrast to the M cluster, the N cluster was further divided into sub-haplogroups, such as A5a, B4a, B4b, B4c, B5^*^, F1a, F1b, N9a, and N9b.

UMAP is a recently developed nonlinear dimensionality reduction algorithm, which has a merit in preserving the topology of the local and global structure^26^. Although UMAP has been previously applied to high-dimensional biological data such as single-cell RNA sequencing^27^, here we applied it to the genomic data of the mtDNA variants. An application of UMAP could classify the subjects into >20 clusters, which was concordant with the pre-defined sub-haplogroups with three letters (Fig. 2c). UMAP could differentiate the sub-haplogroups belonging to the same macrohaplogroup in more detail (e.g., D4a, D4b1, D4b2, D4c, D4e, and D4g belonging to D). On the other hand, we did not find the clear delineation between the M and N clusters, which was observed in the phylogenetic approach and PCA. Several sub-haplogroups with small sample sizes were clustered closely with other macrohaplogroups (e.g., M7c clustered with D macrohaplogroup). These observations could be the potential limitations of UMAP. As each ML classification method had unique advantages to classify mtDNA variations, we would propose a value of applying multiple ML methods to comprehensively visualize haplogroup diversity within a population.

### Distinct characteristics of mtDNA variant structure from nDNA

**Fig. 3:**
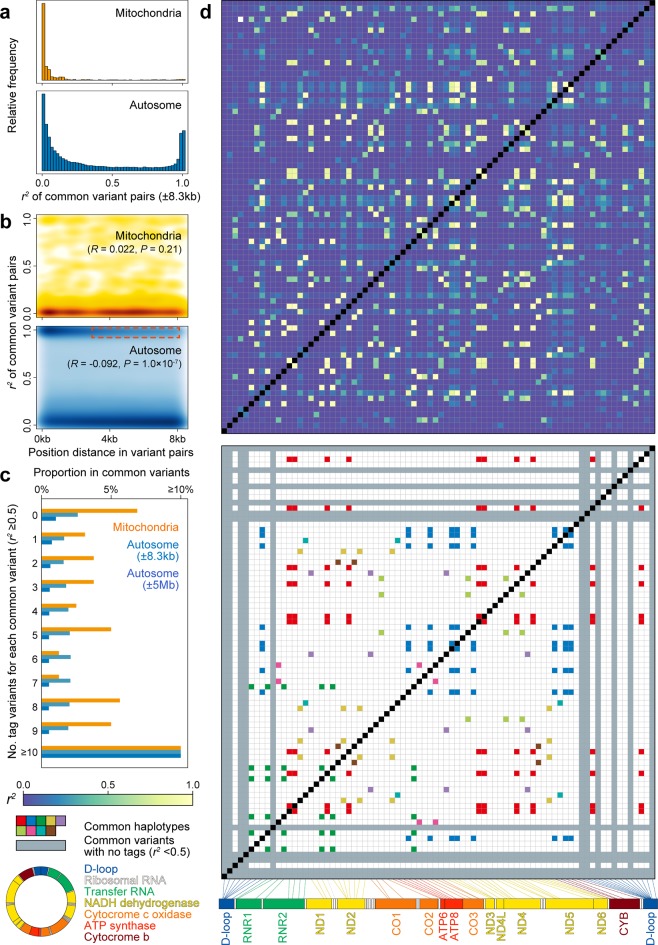
LD structure of the mtDNA variants. LD structure of the common mtDNA variants identified by WGS. **a** Distributions of the haplotype correlations (*r*^2^) and **b** distance-dependent LD decay in the mtDNA and nDNA variants are illustrated in parallel. LD calculation of the nDNA variant pairs was adjusted for the distance corresponding to the mtDNA length (±8.3 kbp, *P* = 0.21 [*R* = 0.022] and *P* = 1.0 × 10^−7^ [*R* = −0.092] for mtDNA and nDNA, respectively). Distance-dependent LD decay in nDNA is highlighted with red. **c** Distributions of the number of the tag variants (*r*^2^ ≥ 0.5) per a common variant. **d** Pairwise LD matrix among the common mtDNA variants (MAF ≥ 5%). In the upper panel, the *r*^2^-values are colored according to the legend. In the lower panel, the variants without any tag variants are highlighted in gray, whereas the variants included in the common haplotypes spanning the entire mtDNA are separately colored as in the legend. Mitochondrial gene positions in mtDNA are indicated in the legend.

Due to lack of recombination and higher mutation rate, LD structure and tag variant distribution in mtDNA are considered to be distinct from those in nDNA, whereas the details have been unclear especially in non-Europeans. Thus, we conducted a comprehensive assessment of these features in Japanese by using the WGS data. In addition to calculate the haplotype correlations (*r*^2^) between common mtDNA variant pairs (MAF ≥ 5%, *n* = 80), we estimated those obtained from the randomly selected autosomal phased haplotypes with adjustment on variant positional differences (±8.3 kbp). Fractions of the variant pairs with high correlations were relatively smaller in mtDNA than in nDNA (2.4% and 20.2% of the variant pairs with *r*^2^ ≥ 0.9 in mtDNA and nDNA, respectively; Fig. 3a).

When we checked distance-dependent decay of LD, we observed clear LD decay in the nDNA variant pairs according to physical distances between the variant pairs, which would reflect LD blocks (*R* = −0.092, *P* = 1.0 × 10^−7^, as highlighted with red; Fig. 3b). However, mtDNA variant showed no distance-dependent LD decay (*R* = 0.022, *P* = 0.21). Although there existed controversial discussions^16,28^, our results did not support the hypothesis of potential recombination events in mtDNA. Lack of recombination and relatively weak LD in mtDNA should propose that the common mtDNA variants are sparsely tagged by the surrounding single-nucleotide polymorphisms (SNPs) when compared with nDNA. The numbers of the tag variants per common variant were smaller in mtDNA than in nDNA, even when the variant distances were adjusted (Fig. 3c). As many as 13.8% of the common mtDNA variants did not have any tag variants with *r*^2^ ≥ 0.5, whereas only 5.0% in nDNA.

Systematic visualization of pairwise LD patterns revealed that mtDNA variants did not constitute LD blocks among neighboring variants (Fig. 3d). On the other hand, we observed multiple common haplotypes spanning the entire mtDNA (*n* = 8), which might have been the consequence of lack of recombination and no distance-dependent LD decay. Interestingly, mtDNA variants without any tag variants were mostly identified in the D-loop region, one of the non-coding but functional regions in mtDNA. By using WGS data, we highlighted the characteristics of the mtDNA variants^28^, which were (i) no distance-dependent LD decay (i.e., absence of LD blocks) and (ii) sparse tagging of the common variants, in non-European population. In addition, we could newly detect existence of the common haplotypes spanning the entire mtDNA.

### No evidence of mtDNA–nDNA genotype association

**Fig. 4:**
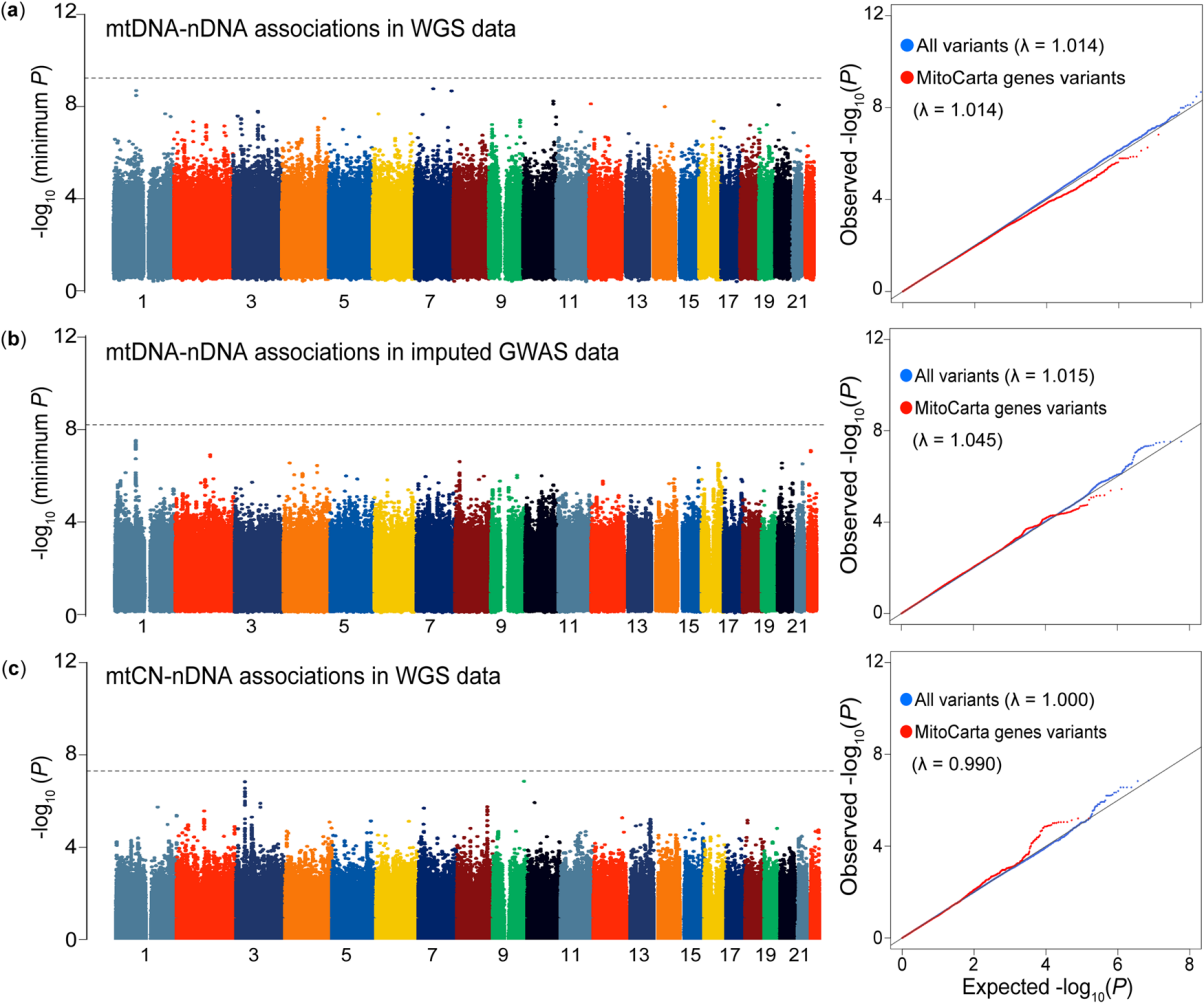
Genome-wide scan of the mtDNA–nDNA (or mtCN-) genotype associations. The plotted *P*-value is the association *P*-value for each analysis. In each panel, a Manhattan plot and a quantile–quantile (QQ) plot are indicated. The *y*-axes of the Manhattan plots in **a** and **b** indicate −log_10_(minimum *P*) at each nDNA variant extracted from the results for all mtDNA variants tested. The horizontal gray lines represent the study-wide significant threshold (*P* < 5.8 × 10^−10^, 6.3 × 10^−9^, and 5.0 × 10^−8^ for **a**, **b**, and **c**, respectively). In the QQ plot, blue dots indicate all the variants and red dots are the variants within ±10k bp of the 1105 mitochondria-related genes in nDNA. **a** The genome-wide mtDNA–nDNA genotype associations obtained from the WGS data (*n* = 1928, mtDNA variants = 86 [MAF ≥ 5%], and nDNA variants = 7,124,343 [MAF ≥ 1%]). **b** The genome-wide mtDNA–nDNA genotype associations obtained from the imputed GWAS data (*n* = 141,552, mtDNA variants = 8 [MAF ≥ 5%], and nDNA variants = 7,402,102 [Rsq ≥ 0.7 and MAF ≥ 1%]). **c** The genome-wide mtCN–nDNA associations obtained from the WGS data (*n* = 1928, nDNA variants = 7,124,343 [MAF ≥ 1%]).

Mitochondrial function is regulated not only by the genes encoded in mtDNA but also by those in nDNA. As these two types of genes confer synergistic biological functions^6^, co-evolution of the genetic variants embedded within them^5^, namely “mtDNA–nDNA genotype association”, has been suggested^17^. Thus, we tested the hypothesis that there might exist preferential transmission between nDNA and mtDNA at a genotype level. To explore footprints of the mtDNA–nDNA genotype association, we conducted a genome-wide scan to assess genotype distribution dependence between mtDNA and nDNA variants using the WGS data. We investigated 86 common mtDNA (MAF ≥ 5%) and the genome-wide 7,124,343 nDNA variants (MAF ≥ 1%), but there was no significant mtDNA–nDNA genotype association when multiple comparisons were considered (*P* = 5.0 × 10^−8^/86 = 5.8 × 10^−10^; Fig. 4a). Even when we focused on the variants located within ±10 kbp of the previously defined nDNA mitochondrial genes (*n* = 1105)^29^, we still did not detect a significant association. In addition, we investigated the associations by using the imputed GWAS data (*n* = 141,552; see details in the next section). We analyzed the association between the 8 mtDNA (MAF ≥ 5%) and the 7,402,102 nDNA variants (Rsq ≥ 0.7 and MAF ≥ 1%), but no significant signals were detected (*P* = 5.0 × 10^−8^/8 = 6.3 × 10^−9^). We neither observed the enrichment of the association signals in the mitochondrial-related gene variants in nDNA (Fig. 4b).

As mtCN of individuals is known to be correlated with mitochondrial functions regulated by the nDNA genes^3,4^, we also investigated the mtCN–nDNA genotype association. Each mtCN of the individual was estimated by the WGS depth. We did not detect any association that satisfied the genome-wide significance threshold (*P* < 5.0 × 10^−8^) and no enrichment of the association signals was observed when we focused on the mitochondria-related gene variants in nDNA (Fig. 4c). Although our WGS study had a relatively larger sample size than the previous ones, we did not find an evidence of mtDNA- (or mtCN-) nDNA genotype association. The mtCN–nDNA association in *TFAM* gene (rs11006126) has been reported^3^. We observed a nominal association with the same directional effect (*P* = 0.018).

### Mitochondrial PheWAS identified abundant trait associations of mtDNA variants

To depict phenotypic landscape of the mtDNA variants on human complex trait, PheWAS utilizing large-scale biobank with deep genotype and phenotype data is necessary. To this aim, we conducted mtDNA PheWAS using the BioBank Japan project, one of the largest biobanks in non-European ancestry^30–33^. After an application of strict quality control filters (see details in Method), we obtained the 44 mtDNA variants (MAF ≥ s0.5%) from the GWAS typing data for the 147,437 Japanese individuals. In addition, we constructed an imputation reference panel of the mtDNA variants based on our WGS data (*n* = 1928). We confirmed imputation accuracy of the reference panel using the cross-validation approach (99.8% of concordance when imputing randomly removed 50% of the genotypes)^34^. We also calculated the minor allele concordance for each MAF bin (0~5, 5~10, 10~20, 20~30, 30~40, 40~50%, respectively). Median concordances were 1.00 for all the bins and means were 0.84, 0.96, 0.93, 0.95, 1.00, and 0.98 for each bin. We then imputed the mtDNA variants of the individuals with the GWAS data. After an application of stringent post-imputation variant filtering (MAF ≥ 0.5% and imputation score info ≥ 0.7), we obtained genotype dosages of 206 mtDNA variants. Using the imputed dosages of the GWAS individuals, we conducted mitochondrial PheWAS to comprehensively reveal the association between genotype and 99 phenotypes (46 complex diseases, 49 quantitative traits (quantitative trait loci), and 4 drinking and smoking habits; Supplementary Data 3) with robust adjustment for potential population stratification by including the geographic regions of participants and the top 20 PCs as covariates.

**Fig. 5:**
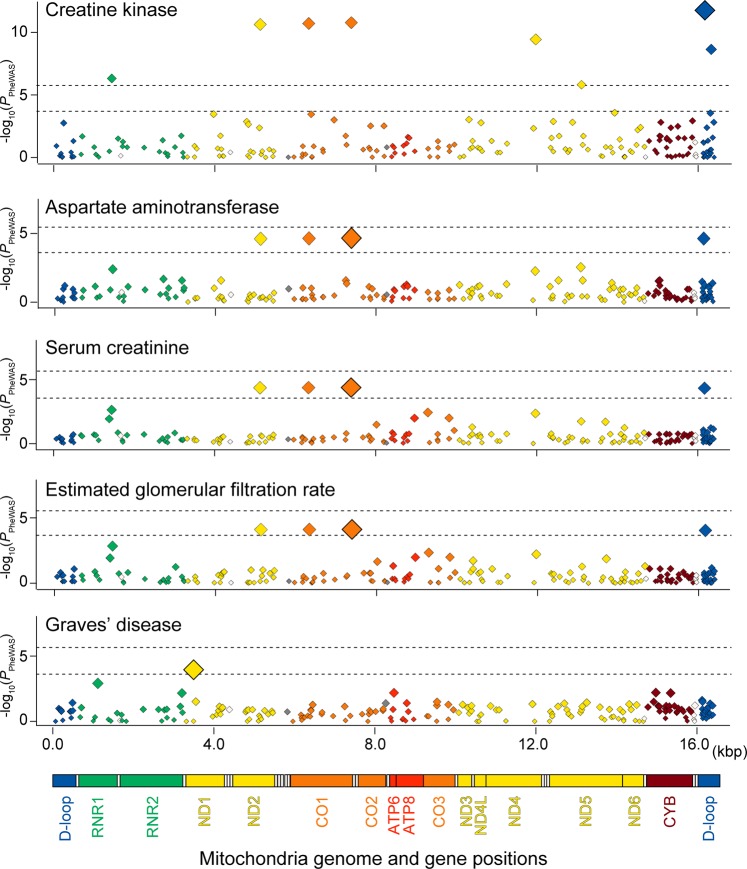
Mitochondrial regional plots indicating genotype–phenotype associations identified by PheWAS. Regional association plots of the entire mtDNA illustrating the genotype–phenotype associations identified by the mitochondrial PheWAS in the Japanese population (*n* = 147,310). *Y*-axes represent –log_10_(*P*) of the associations of the variants and *x*-axes represent the base pair positions in mtDNA. An upper horizontal bar in each plot represents the study-wide significance threshold of *P* = 2.5 × 10^−6^, considering multiple comparisons of both the numbers of the variants and phenotypes. A lower horizontal bar in each plot represent the study-wide significance threshold of *P* = 2.4 × 10^−4^, considering multiple comparisons of the number of the variants. Mitochondrial gene positions are indicated in the bottom panel.

We observed a significant association of the mtDNA variant with one of the biochemical quantitative traits, creatine kinase, which satisfied the study-wide significance threshold considering the multiple comparisons of the number of the variants and phenotypes (*P* < 0.05/206/99 = 2.5 × 10^−6^; Table 1 and Fig. 5). The low-frequency variant at the D-loop region, MT:16168:C:T, showed the most significant association (MAF = 0.007, *P* = 1.7 × 10^−12^). This variant was in strong LD with other three variants, which showed similar associations (MT:5127:A:G at *MT-ND2*, and MT:6332:A:G and MT:7389:T:C at *MT-CO1*; *r*^2^ > 0.86, |*D*′| = 1), and these four low-frequency variants corresponded to the B4f sub-haplogroup.

**Table 1:** Phenome-wide association study results of the mtDNA variants.

Trait category	Trait	Total (case, control)	Variant	Position^**a**^	Ref/Alt	Gene	Annotation	Frequency^**b**^	Info score	Effect size^**c**^	*P*
mtDNA variants with *P* < 0.05/206/99 = 2.5 × 10^−6 d^											
Oher biochemical quantitative trait	Creatine kinase	93,664	MT:16168:C:T	16,168	C/T	D-loop	Upstream	0.007	0.830	0.156 (0.022)	1.7 × 10^**−**12^
mtDNA variants with *P* < 0.05/206 = 2.4 × 10^−4 e^											
Liver-related quantitative trait	AST	118,177	MT:7389:T:C	7389	T/C	MT-CO1	Missense	0.007	0.990	0.075 (0.018)	2.2 × 10^**−**4^
Kidney-related quantitative trait	Serum creatinine	122,745	MT:7389:T:C	7389	T/C	MT-CO1	Missense	0.007	0.990	−0.072 (0.017)	4.2 × 10^**−**5^
Kidney-related quantitative trait	eGFR	122,747	MT:7389:T:C	7389	T/C	MT-CO1	Missense	0.007	0.990	0.069 (0.017)	7.7 × 10^**−**5^
Immune-related disease	Graves’ disease	(1769, 139,856)	MT:3497:C:T	3497	C/T	MT-ND1	Missense	(0.046, 0.033)	1.000^f^	1.30 (1.14–1.48)	1.1 × 10^**−**4^

*Alt* alternative allele, *AST* aspartate aminotransferase, *eGFR* estimated glomerular filtration rate, *Ref* reference allele.

^a^Base pair positions in hg19.

^b^Frequencies of alternative alleles. Those in cases and controls are indicated separately with parenthesis for the binary traits.

^c^Odds ratios and 95% confidence intervals for the binary traits, and betas and SEs for the quantitative traits.

^d^The significance threshold considering multiple comparisons of the number of the phenotypes and variants (*n* = 99 and 206, respectively).

^e^The significance threshold considering multiple comparisons of the number of the variants (*n* = 206).

^f^The variants that were directly genotyped by the microarray, whereas the other variants were imputed.

Association signals satisfying the significant threshold considering multiple comparisons of the variant numbers were observed in additional four phenotypes (*P* < 0.05/206 = 2.4 × 10^−4^). MT:7389:A:G at *MT-CO1* (and other three variants in strong LD as mentioned above) showed associations with the kidney function measurements of serum creatinine (*P* = 4.2 × 10^−5^) and estimated glomerular filtration rate (*P* = 7.7 × 10^−5^). These variants represented by B4f also showed pleiotropic associations with the liver function measurement of aspartate aminotransferase (MT:7389:T:C at *MT-CO1*, *P* = 2.2 × 10^−5^). In addition, we identified associations with immune-related trait such as Graves’ disease, an autoimmune thyroiditis with hyperthyroidism (MT:3497:C:T at *MT-ND1*, *P* = 1.1 × 10^−4^). Although MT:3497:C:T was a low-frequency variant in Japanese and 1KG EAS (MAF = 0.036 and 0.016, respectively), this variant was not observed in the other 1KG populations, suggesting the disease risk specific to east Asian populations. These results clearly demonstrated abundant genotype–phenotype associations embedded in the mtDNA.

Previous studies suggested mtDNA genetics risk on late-onset human clinical phenotypes^15,17,35^. In addition to validation of the previously suggested associations with renal functions, our study provided novel phenotypic associations in creatine kinase, an immune-related disease, and a liver function-related trait. Creatine kinase is an enzyme highly expressed in tissues that rapidly consume energy such as skeletal muscle and brain, which are frequently affected in mitochondrial diseases. Creatine kinase is suggested as a potential biomarker to reflect mitochondrial dysfunction^36^, for which genetic and phenotypic link with mitochondria should provide novel biological insights. Patients affected with mitochondrial diseases often confer endocrine dysfunction including thyroid hormones^37^, whereas the biological link with a liver function-related trait is elusive. Our study would provide novel insights into the genetic risk of the mtDNA variants on human complex diseases.

## Discussion

Although it has been known that mitochondria play a crucial role in homeostasis, there are few mitochondrial genome-wide studies considering population stratification. Therefore, our comprehensive study of mtDNA should enhance the genetic and biological knowledge about mitochondria. Through the genetic and phenotypic analysis of the mtDNA based on the variants obtained from the large-scale WGS and GWAS data of the Japanese individuals, our study successfully elucidated the population-specific mtDNA structure and their pleiotropic associations with the human complex traits.

Our study highlights the following novel features. First, we could define the high-resolution spectra of the haplogroups, which ranged from one to nine letters. The frequency spectra of the haplogroups were population-specific to Japanese and even heterogeneous among the geographic regions within Japan. This should propose an importance of WGS-based cataloging of the detailed haplogroups among diverse populations. Second, by applying the latest nonlinear ML dimensionality reduction method of UMAP, we could deconvolute the finer individual classifications corresponding to the high-digit sub-haplogroups with three letters than the traditional linear ML method. This result expands application of the nonlinear ML method to extract novel human genome features without prior knowledge. Third, mtDNA variants had distinct features from those of nDNA: (i) no distance-dependent LD decay, (ii) sparse common variant tagging, and (iii) multiple common haplotypes spanning the entire mtDNA. Especially, mtDNA variants without common tagging variants were mostly observed in the D-loop region, possibly due to the high mutation rate and recurrent mutations in this region^22^. Considering that not all the mtDNA common variants were genotyped (or tagged) by the current SNP microarrays, these features propose the necessity of cataloging the variant lists to efficiently tag the mtDNA variations in diverse ethnicity. Fourth, we did not find an evidence of mtDNA- (or mtCN-) nDNA genotype association. Although mtDNA–nDNA associations have been reported in an animal experiment and a human gene expression study^5,6^, further investigation would be necessary to elucidate their genotype-level associations.

Finally, Biobank-driven mitochondrial PheWAS revealed abundant mtDNA genetic risks on a variety of human complex traits, including newly identified risks on creatine kinase and an immune-related trait (Graves’ disease). Our PheWAS was able to detect pleiotropic effects of the low-frequency mtDNA variants corresponding to the specific sub-haplogroup. As the majority of the haplogroups was rare and difficult to be imputed with high accuracy, a haplogroup-based PheWAS was not conducted in our study. The detected variants had relatively moderate effect sizes when compared with the pathogenic rare mtDNA mutations causing congenital mitochondrial diseases, suggesting their modifier roles on the phenotypes^38^. Our study demonstrated a successful imputation of the mtDNA variants by using the large-scale WGS as a reference panel. However, a part of the common mtDNA variants were not imputed due to lack of the genotyped tag SNPs and it would also be difficult to fine-map the causal variants when the identified risk variant belonged to the common haplotypes spanning the entire mtDNA. Further risk fine-mapping efforts including custom-design of the variant list on the microarrays, *trans*-ethnic comparisons, a replication study investigating our PheWAS findings, and functional validation assays would be warranted.

In conclusion, through the large-scale WGS and PheWAS of mtDNA, our study could comprehensively elucidate the genetic and phenotypic landscapes of mtDNA in the Japanese population.

## Method

### Subjects

All the subjects enrolled in the WGS and the PheWAS were the BioBank Japan Project participants. BioBank Japan Project is a multi-institutional hospital-based study that collected peripheral blood DNA, serum, and clinical information from the participants affected with any of the 47 target diseases^30^. The participants provided written informed consent as approved by the ethical committees of the Institute of Medical Science, the University of Tokyo. The details of the characteristics of individuals were described in elsewhere^30,39,40^. This study was approved by the ethical committee of Osaka University Graduate School of Medicine.

### Mitochondrial DNA variant calling from the WGS data

WGS was conducted by using HiSeq 2500 with 160 bp and 125 bp pair end (*n* = 1269) or HiSeq X with 150 bp pair end (*n* = 659), which achieved high-depth on autosomal chromosomes (20–35×) as described elsewhere^18^. In this study, we realigned the sequenced reads on the human reference genome sequences of all the chromosomes and mitochondria. We extracted the uniquely mapped reads on the mitochondrial region to minimize the contamination of nuMTs^19^. In brief, the sequence reads were trimmed to remove Illumina adapter by Trimmomatic (version 0.36). The trimmed reads were mapped by BWA-MEM (version 0.7.15) on human reference genome sequence GRCh37 including rCRS (NC_012920.1) as mitochondrial reference genome sequence. After sorting by Samtools (version 1.4.1), mitochondrial regions (MT index) were extracted. Duplicated reads were removed using picard (version 2.9.2). Base recalibration was done by using GATK (version 3.7.0). Individual variant calling was performed using GATK HaplotypeCaller in the setting of ploidy 1 to assess homoplasmy. Multi-sample joint-calling of the variants were also conducted by GATK GenotypeGVCFs. Finally, manual filtering was done according to GATK best practice pipeline because of an insufficient number of variants to perform variant quality score recalibration^41^. The Ti/Tv ratio was estimated using bcftools (version 1.4.1).

### Assignment protocol of the haplogroups based on the mtDNA variants

Using the detected mtDNA variants as an input, each individual was classified into a mitochondrial haplogroup by using HaploGrep (v2.1.14), an unsupervised clustering method based on Phylotree17^24^. The first letter of the haplogroup was defined as a marohaplogroup (e.g., “M”). From the second to the ninth letters of the haplogroups were decided as sub-haplogroups 1 to 8 (e.g., “M9”, “M9a”, “M9a1”,…, “M9a1a1c1a”). For comparison, we parallelly defined haplogroups of the 2504 individuals from the 1KG phase 3 in the same way.

### Unsupervised classification of the samples using the ML methods

We adopted the three unsupervised mtDNA variant classification methods as follows: (1) phylogenetic analysis, (2) PCA as a linear dimensionality reduction technique, and (3) UMAP as a nonlinear dimensionality reduction technique. In the phylogenetic analysis, the variant list of the individual was converted to the FASTA format by using bcftools (version 1.4.1), realigned by using MUSCLE (version 3.8.31), and switched to the Phylip format. Phylogenetic tree was inferred using the maximum-parsimony method of PHYLIP (version 3.697) and figured using FigTree (version 1.4.3). We conducted PCA and UMAP using Scikit-learn of python (version 3.7) with the haploid genomes as inputs. PCA was calculated to obtain the top 20 components. The setting in applying UMAP was n_neighbors = 100 and min_dist = 0.99.

### mtDNA variant characterization by LD structure assessment

To evaluate LD structure in mtDNA, we calculated pairwise haplotype correlations, equivalent to the original definition of the LD metric of *r*^2^, of the common mtDNA variants (MAF ≥ 5%). As a reference, we calculated pairwise LD of the randomly selected identical numbers of the phased autosomal haplotypes in a genome-wide manner. We adjusted the variant distance differences by restricting the autosomal variant pairs within the distances corresponding to those in mtDNA (±8.3 kbp). As for the tag variant identification, *r*^2^-values of the common variants with all the neighboring autosomal variants (±5 Mbp) were calculated. Common haplotypes consisting of the mtDNA variants were defined by selecting sets of the mtDNA variant pairs with strong LD (*r*^2^ ≥ 0.8).

### Assessment of mtDNA–nDNA and mtCN–nDNA genotype associations

We evaluated genetic correlations between mtDNA and nDNA variants (or mtCN). Autosomal DNA variants with call rate ≥ 0.99, MAF ≥ 1%, and Hardy–Weinberg equilibrium *P*-value ≥ 1.0 × 10^−6^ were chosen from the previous WGS study (*n* = 1928)^18^. MtDNA variants with MAF ≥ 5% in the same set of individuals were selected. The mtCN of each individual was quantified by the formula, as descried elsewhere^5^:

$${\mathrm{mtDNA}\,\mathrm{copy}\,\mathrm{number}} = \frac{{\mathrm{Average}\,\mathrm{mtDNA}\,\mathrm{depth}}}{{\mathrm{Average}\,\mathrm{autosomal}\,\mathrm{depth}}} \times 2$$

Both of autosomal and mitochondrial average depths were extracted by the VCFtools (version 0.1.14) from the VCF files. By averaging the mtCN for each autosomal chromosome, the mean mtCN across all the chromosomes was calculated^5,42^. Regarding mtDNA–nDNA genotype association, logistic regression by PLINK (version 1.90b4.4) was performed using nDNA variants as explanatory variables. Regarding mtCN–nDNA genotype association, linear regression by PLINK (version 1.90b4.4) was conducted using nDNA variants as explanatory variables. To handle potential confounding factors, we included the top 20 principal components (PCs) of the autosomal variants and the WGS run batches as covariates. Especially, to examine genetic correlation with the nDNA variants in the mitochondria-related genes, the variants within ±10 kbp of the 1105 autosomal genes registered in mitoCarta2.0 were selected^29^. We adopted a genome-wide significance threshold considering the multiple comparison of the mtDNA variants for mtDNA–nDNA genotype association (*P* < 5.0 × 10^−8^/86 = 5.8 × 10^−10^) and the typical genome-wide significance threshold for mtCN–nDNA genotype association (*P* < 5.0 × 10^−8^)^43^. Next, we extracted autosomal DNA genotype dosages with Rsq ≥ 0.7 and MAF ≥ 1% from previous imputed GWAS data, and mtDNA variants with MAF ≥ 5% of the same individuals were selected (*n* = 141,552). Regarding mtDNA–nDNA genotype association, a logistic regression by PLINK (version 2.00a2LM) was performed in the same way. We included age, sex, the top 20 PCs, genotype microarray platforms, and geographical regions of participants as covariates. We adopted a genome-wide significance threshold considering the multiple comparison of the mtDNA variants (*P* < 5.0 × 10^−8^/8 = 6.3 × 10^−9^).

### Mitochondrial PheWAS

We genotyped the BioBank Japan Project participants using the Illumina HumanOmniExpressExome BeadChip or a combination of the Illumina HumanOmniExpress and HumanExome BeadChips. Normalized probe intensities were extracted for all the individuals passing standard laboratory quality control thresholds and genotypes were called using optiCall (version 0.7.0). We specified the -MT option to call mitochondrial variants and used the default settings. Genotypes with an individual posterior probability lower than 0.7 were defined as unknown. We excluded the individuals with (i) closely related individuals identified by identity-by-descent analysis or (ii) non-East Asian outliers identified by PCA of autosomal variants. Then, we applied the quality control criteria for mtDNA variants, excluding the individuals with sample call rate < 0.9, the variants with call rate < 0.99, or the variants with the concordance rate < 0.99 between the SNP array and the WGS data (*n* = 1446).

We conducted mtDNA variant imputation of the BioBank Japan Project GWAS data. We constructed the WGS-based mtDNA imputation reference panel of the Japanese population (*n* = 1928). The imputation accuracy of the constructed mtDNA imputation reference panel was evaluated by a cross-validation method as described previously^38^. The minor allele concordances of the sequence and imputed variants were calculated for each of the MAF bins separately (0~5, 5~10, 10~20, 20~30, 30~40, 40~50%, respectively). We selected the variants with MAF ≥ 0.5% in the GWAS genotype data, filled missing genotype by Eagle (v2.4.1), and imputed by IMPUTE2 (version 2.3.2) with the options of –m 0 and –chrX using the constructed mtDNA imputation reference panel. We applied post-imputation QC filtering of the variants (MAF ≥ 0.5% and imputation score info ≥ 0.7) for the PheWAS.

We curated the phenotype record of the disease status and clinical values for the BioBank Japan Project participants (*n* = 147,310). The diseases composed of the five major categories (immune related [*n* = 10], metabolic and cardiovascular [*n* = 10], cancers [*n* = 13], ophthalmologic [*n* = 2], and other diseases [*n* = 11]). The quantitative traits composed of the ten categories (anthropometric [*n* = 2], metabolic [*n* = 6], protein [*n* = 4], kidney related [*n* = 4], electrolyte [*n* = 5], liver related [*n* = 5], other biochemical [*n* = 6], hematological [*n* = 13], blood pressure [*n* = 4], and behavior [*n* = 4]). A group of individuals not affected by the disease under scope was used as a control group in the analysis. Then, we performed the logistic regression analyses for the 46 diseases and the 2 binary traits in behavior category, with the adjustment for age, sex, the top 20 PCs, geographic regions, and the genotype microarray platforms as covariates. For the 51 quantitative traits, we performed the linear regression analyses on the rank-normalized residuals after regressing out using age, sex, the top 20 PCs, and the trait-specific covariates as described elsewhere^31,32,44,45^. We additionally included geographic regions and the genotype microarray platforms as covariates in linear regression. Association studies were done with a glm() function implemented in R statistical software (version 3.4.0) using the imputed dosage data.

### Statistics and reproducibility

Software and database used for the data analysis of this study are as follows: bcftools (https://samtools.github.io/bcftools/bcftools.html), BioBank Japan Project (https://biobankjp.org/english/index.html), BWA (http://bio-bwa.sourceforge.net/), Eagle (https://data.broadinstitute.org/alkesgroup/Eagle/), FigTree (http://tree.bio.ed.ac.uk/software/figtree/), GATK (https://software.broadinstitute.org/gatk/), HaploGrep (https://github.com/seppinho/haplogrep-cmd), IMPUTE2 (https://mathgen.stats.ox.ac.uk/impute/impute_v2.html), MitoCarta2.0 (https://www.broadinstitute.org/scientific-community/science/programs/metabolic-disease-program/publications/mitocarta/mitocarta-in-0), mtSNP database (http://mtsnp.tmig.or.jp/mtsnp/index_e.shtml), MUSCLE (https://www.drive5.com/muscle/), optiCall (https://opticall.bitbucket.io/), PHYLIP (http://evolution.genetics.washington.edu/phylip.html), Picard (https://broadinstitute.github.io/picard/), PLINK (https://www.cog-genomics.org/plink2), R (https://www.r-project.org/), rCRS (https://www.mitomap.org/MITOMAP/HumanMitoSeq), The 1000 Genomes Project (http://www.internationalgenome.org/), Trimmomatic (http://www.usadellab.org/cms/?page=trimmomatic), VCFtools (http://vcftools.sourceforge.net/), and 3.5KJPNv2 (https://ijgvd.megabank.tohoku.ac.jp/).

### Reporting summary

Further information on research design is available in the Nature Research Reporting Summary linked to this article.

## Supplementary information

Supplementary Data 1

Supplementary Data 2

Supplementary Data 3

Description of Additional Supplementary Files

Supplementary Information

Reporting Summary

## Data Availability

GWAS and WGS data used in this study are available at the National Bioscience Database Center (NBDC) Human Database (Accession codes: hum0014).

## Code availability

No previously unreported custom computer code or mathematical algorithm was used to generate results central to the conclusions.
