# Supplementary material for: Genetic and phenotypic landscape of the mitochondrial genome in the Japanese population
Source: Commun Biol. 2020 Mar 5;3:104. doi: 10.1038/s42003-020-0812-9 (PMC7058612; doi:10.1038/s42003-020-0812-9)
Supplement: Supplementary file 4 — Description of Additional Supplementary Files [file 42003_2020_812_MOESM4_ESM.docx]

**Description of Supplementary Data**

**Supplementary Data 1:** List of the mtDNA variants identified in the WGS data of Japanese.

**Supplementary Data 2:** Geographical distribution of the frequency spectra of the haplogroups in the Japanese population.

**Supplementary Data 3:** Descriptions of the clinical phenotypes used in the phenome-wide association study.
