# Supplementary material for: Genetic and phenotypic landscape of the mitochondrial genome in the Japanese population
Source: Commun Biol. 2020 Mar 5;3:104. doi: 10.1038/s42003-020-0812-9 (PMC7058612; doi:10.1038/s42003-020-0812-9)
Supplement: Supplementary file 5 — Supplementary Information [file 42003_2020_812_MOESM5_ESM.pdf]

Genetic and phenotypic landscape of the mitochondrial genome in the Japanese population

Supplementary Figure 1. The results of the mitochondrial genome sequencing of the Japanese population.

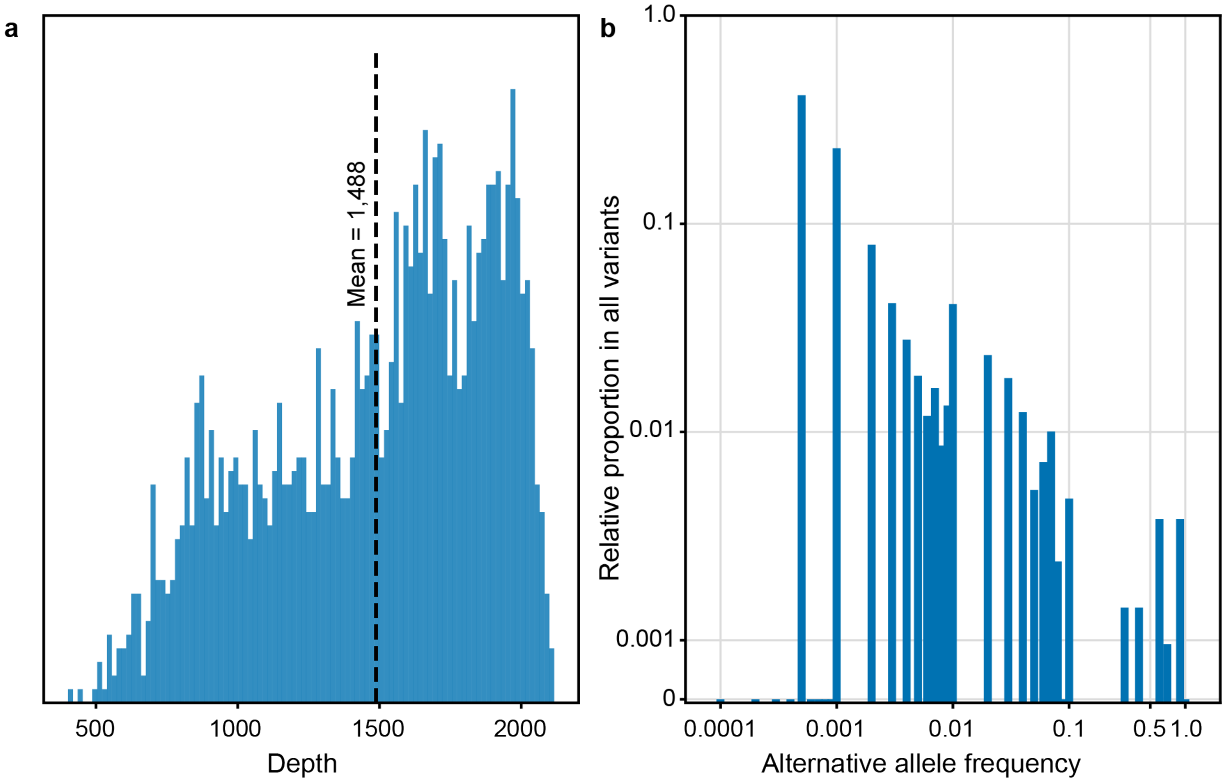

(**a**) The distribution of the depth of each sample of the mitochondrial genome sequencing. The vertical dash line represents mean depth. (**b**) Alternative allele frequency spectrum of the variants identified in the whole mitochondrial genome sequence analysis of the Japanese population.

Supplementary Figure 2. The comparison of the alternative allele frequency spectra with other mtDNA variant databases of the Japanese population.

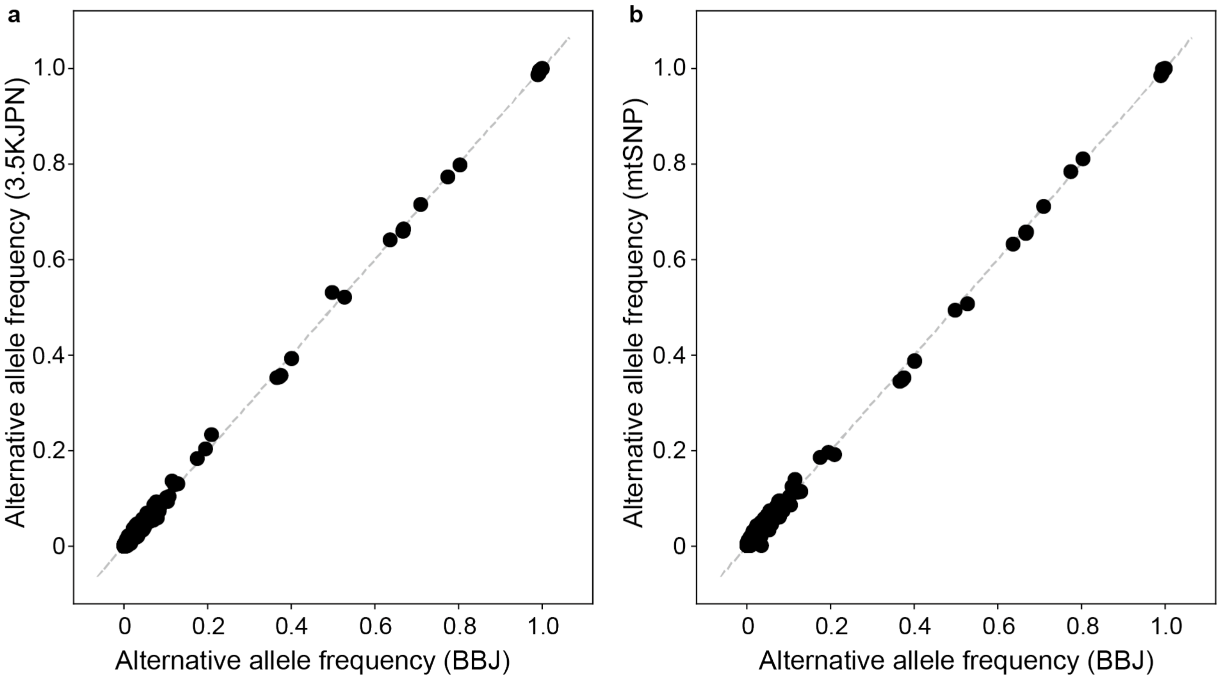

The comparison of the alternative allele frequency spectra of the mtDNA variants identified in the mitochondrial genome sequencing with (**a**) 3.5KJPN and (b) GiiB-JST mtSNP databases, respectively.

Supplementary Figure 3. Mutational spectrum of the mtDNA variants.

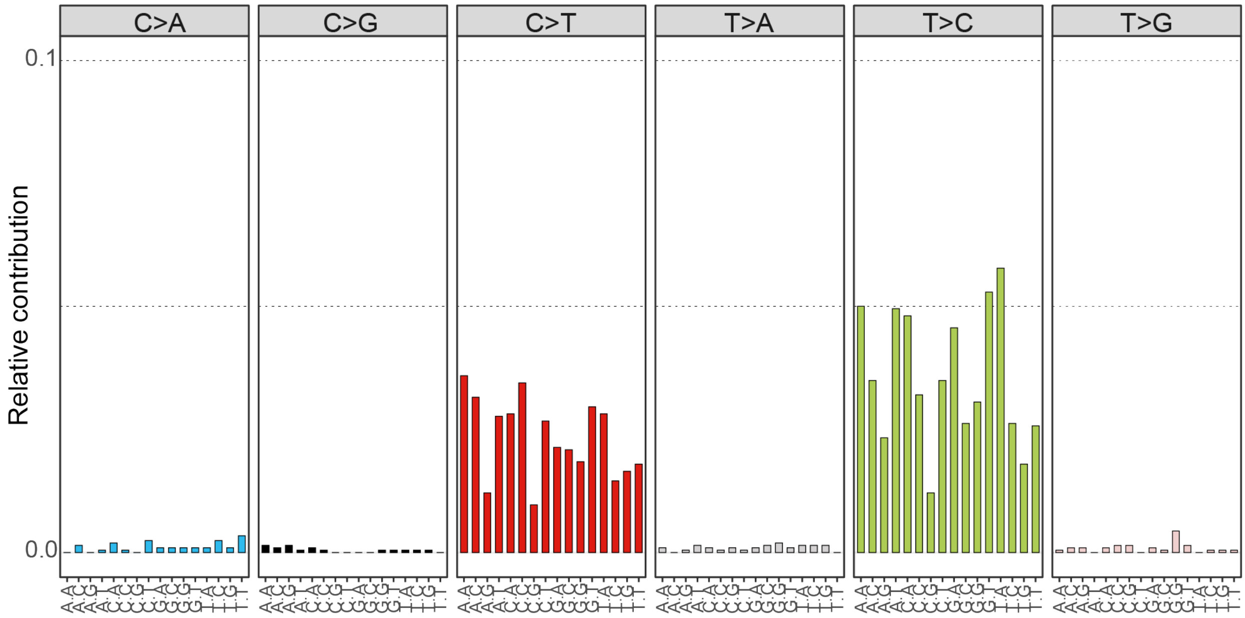

Mutational spectrum of the mtDNA variants identified by using the mitochondrial genome sequencing of the Japanese individuals.

Supplementary Figure 4. The geographical region map of Japan.

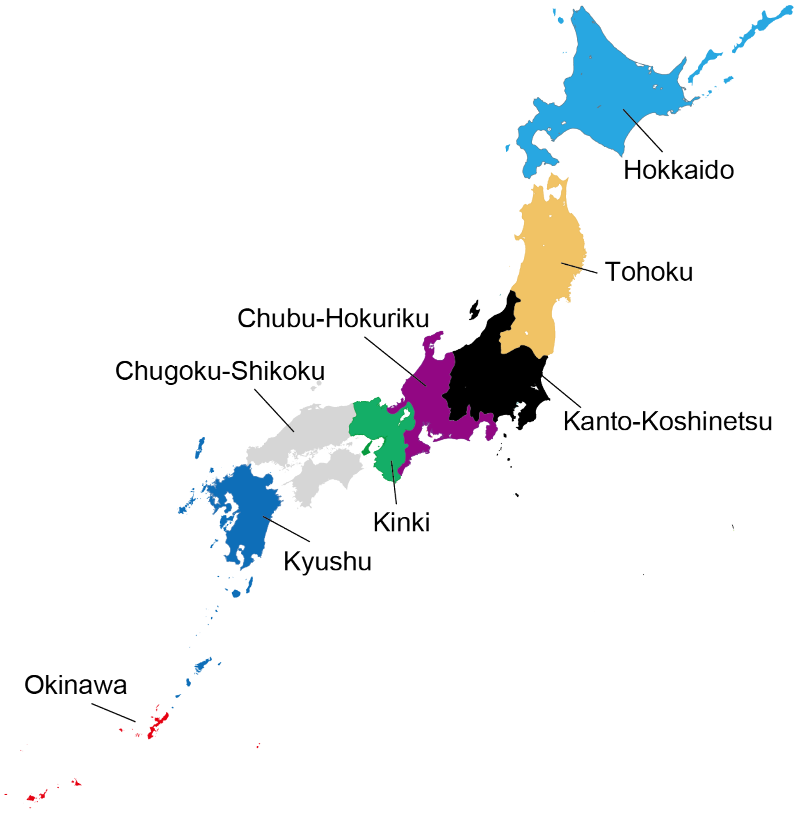

Supplementary Figure 5. Frequency distribution of the sub-haplogroup per geographical region in Japan.

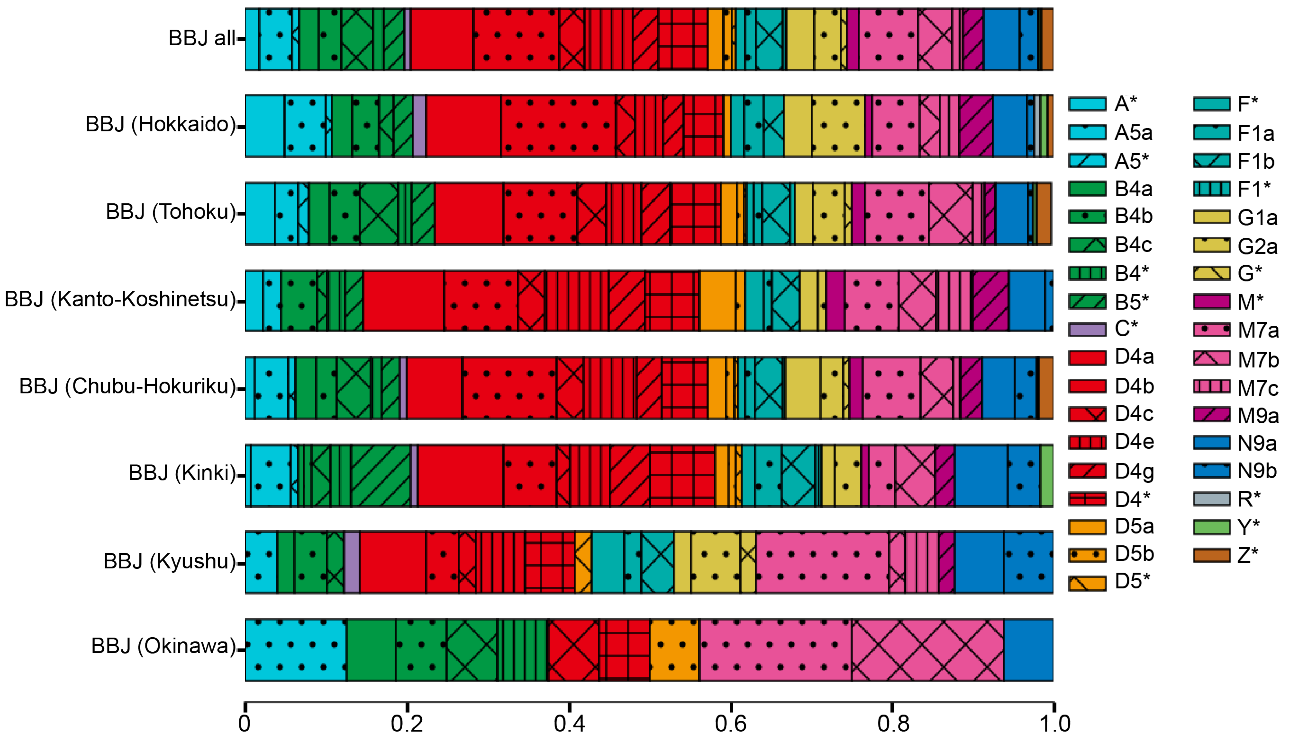

The stacked bar plot of the frequency of sub-haplogroup (3 letters) per geographical region in Japan. The sub-haplogroups less than 1% were assembled in the previous sub-haplogroups. (i.e. “A*”, “A5*”, “B4*”, “B5*”, “C*”, “D4*”, “D5*”, “F*”, “F1*”, “G*”, “M*”, “R*”, “Y*”, and “Z*”)

Supplementary Figure 6. The pairwise plots of the top 10 PCs in the mtDNA variants.

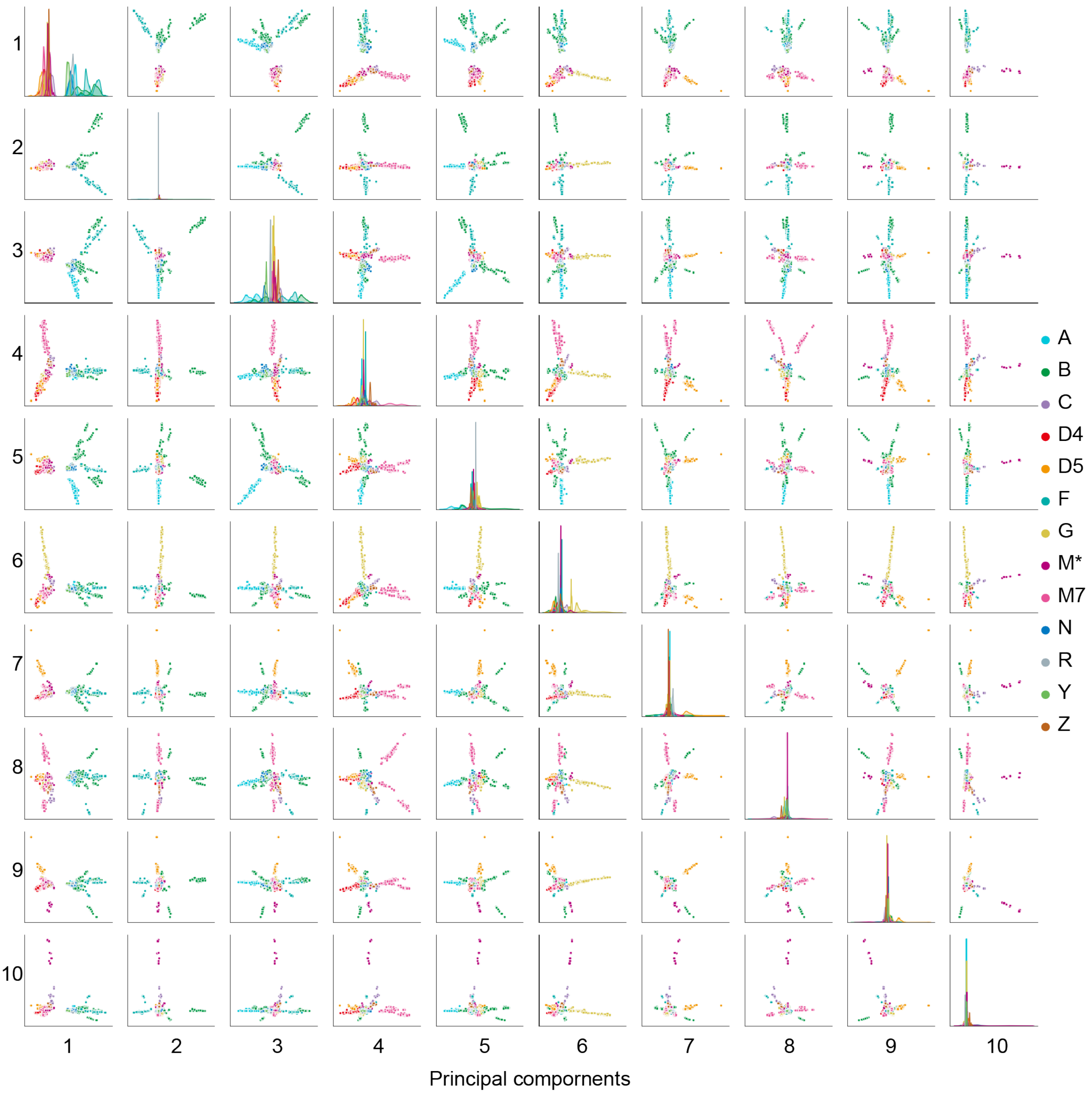

The x- and y-axes represent principal components of the mtDNA variants of the Japanese individuals. Color annotation was based on the haplogroup classifications.
