# Supplementary material for: Genetic and phenotypic landscape of the mitochondrial genome in the Japanese population
Source: Commun Biol. 2020 Mar 5;3:104. doi: 10.1038/s42003-020-0812-9 (PMC7058612; doi:10.1038/s42003-020-0812-9)
Supplement: Supplementary file 6 — Reporting Summary [file 42003_2020_812_MOESM6_ESM.pdf]

nature research

Corresponding author(s): Yukinori Okada

Last updated by author(s): Jan 20, 2020

Reporting Summary

Nature Research wishes to improve the reproducibility of the work that we publish. This form provides structure for consistency and transparency in reporting. For further information on Nature Research policies, see Authors & Referees and the Editorial Policy Checklist.

Statistics

For all statistical analyses, confirm that the following items are present in the figure legend, table legend, main text, or Methods section.

n/a	Confirmed	
☐	☒	The exact sample size (*n*) for each experimental group/condition, given as a discrete number and unit of measurement
☐	☒	A statement on whether measurements were taken from distinct samples or whether the same sample was measured repeatedly
☐	☒	The statistical test(s) used AND whether they are one- or two-sided<br>*Only common tests should be described solely by name; describe more complex techniques in the Methods section.*
☐	☒	A description of all covariates tested
☐	☒	A description of any assumptions or corrections, such as tests of normality and adjustment for multiple comparisons
☐	☒	A full description of the statistical parameters including central tendency (e.g. means) or other basic estimates (e.g. regression coefficient) AND variation (e.g. standard deviation) or associated estimates of uncertainty (e.g. confidence intervals)
☐	☒	For null hypothesis testing, the test statistic (e.g. *F*, *t*, *r*) with confidence intervals, effect sizes, degrees of freedom and *P* value noted<br>*Give P values as exact values whenever suitable.*
☒	☐	For Bayesian analysis, information on the choice of priors and Markov chain Monte Carlo settings
☒	☐	For hierarchical and complex designs, identification of the appropriate level for tests and full reporting of outcomes
☐	☒	Estimates of effect sizes (e.g. Cohen's *d*, Pearson's *r*), indicating how they were calculated

Our web collection on statistics for biologists contains articles on many of the points above.

Software and code

Policy information about availability of computer code

Data collection	No software was used.
Data analysis	We used publicly available software for the data analysis; Trimmomatic (version 0.36), BWA-MEM (version 0.7.15), Samtools (version 1.4.1), picard (version 2.9.2), GATK (version 3.7.0), bcftools (version 1.4.1), HaploGrep (v2.1.14), MUSCLE (version 3.8.31), PHYLIP (version 3.697), FigTree (version 1.4.3), Scikit-learn of python (version 3.7), VCFtools (version 0.1.14), PLINK (version 1.90b4.4) , Eagle (v2.4.1), IMPUTE2 (version 2.3.2), and R statistical software (version 3.4.0).

For manuscripts utilizing custom algorithms or software that are central to the research but not yet described in published literature, software must be made available to editors/reviewers. We strongly encourage code deposition in a community repository (e.g. GitHub). See the Nature Research guidelines for submitting code & software for further information.

Data

Policy information about availability of data

All manuscripts must include a data availability statement. This statement should provide the following information, where applicable:

- Accession codes, unique identifiers, or web links for publicly available datasets
- A list of figures that have associated raw data
- A description of any restrictions on data availability

GWAS and WGS data of the BBJ subjects is available at the NBDC Human Database (Research ID: hum0014).

Field-specific reporting

Please select the one below that is the best fit for your research. If you are not sure, read the appropriate sections before making your selection.

☒ Life sciences ☐ Behavioural & social sciences ☐ Ecological, evolutionary & environmental sciences

For a reference copy of the document with all sections, see nature.com/documents/nr-reporting-summary-flat.pdf

Life sciences study design

All studies must disclose on these points even when the disclosure is negative.

Sample size	Clinical information and genotype data were obtained from BioBank Japan project7, which is a prospective biobank that collaboratively collected DNA and serum samples from 12 medical institutions in Japan and recruited approximately 200,000 participants. Of them, we included individuals with genotype (GWAS or WGS) and basic phenotype data.
Data exclusions	Regarding WGS data, we applied the previously quality controlled samples as described elsewhere. Regarding GWAS data, we excluded individuals with the age under 18, low call rate in genotyping (< 98%), closely related (PI_HAT < 0.125), and ancestry other than Japanese (based on PCA plot) as described elsewhere.
Replication	We did not conduct the replication since no other Japanese WGS and GWAS data with available phenotype data with similar sample sizes existed.
Randomization	We did not any randomization.
Blinding	We did not apply blinding of the samples.

Reporting for specific materials, systems and methods

We require information from authors about some types of materials, experimental systems and methods used in many studies. Here, indicate whether each material, system or method listed is relevant to your study. If you are not sure if a list item applies to your research, read the appropriate section before selecting a response.

Materials & experimental systems

n/a	Involved in the study	
☒	☐	Antibodies
☒	☐	Eukaryotic cell lines
☒	☐	Palaeontology
☒	☐	Animals and other organisms
☐	☒	Human research participants
☒	☐	Clinical data

Methods

n/a	Involved in the study	
☒	☐	ChIP-seq
☒	☐	Flow cytometry
☒	☐	MRI-based neuroimaging

Human research participants

Policy information about studies involving human research participants

Population characteristics	The detailed information of participants is summarized in Supplementary Table 3.
Recruitment	BioBank Japan project collaboratively collected DNA and serum samples from 12 medical institutions in Japan and recruited approximately 200,000 participants with the diagnosis of at least one of 47 diseases, mainly of Japanese ancestry.
Ethics oversight	All the participants provided written informed consent approved from ethics committees of RIKEN Center for Integrative Medical Sciences, and the Institute of Medical Sciences, the University of Tokyo.

Note that full information on the approval of the study protocol must also be provided in the manuscript.
